# Supplementary material for: Patterns of Intron Gain and Loss in Fungi
Source: PLoS Biol. 2004 Nov 30;2(12):e422. doi: 10.1371/journal.pbio.0020422 (PMC532390; doi:10.1371/journal.pbio.0020422)
Supplement: Table S1 — Also available at http://genes.mit.edu/NielsenEtAl/. (4.3 MB ZIP). [file pbio.0020422.st001.zip › NielsenEtAl/html/427.html]

AN1454.1.NCU09142.1.MG04771.1.FG00428.1


```
 CLUSTAL W (1.82) Multiple Sequence Alignments - Introns Inserted


Sequence 1: NCU09142.1	697 aa
Sequence 2: FG00428.1	684 aa
Sequence 3: MG04771.1	695 aa
Sequence 4: AN1454.1	689 aa
Alignment Length: 723 aa
Number Identitical Residues: 290 aa
Alignment Score (without introns) 15276


MG04771.1 	-----MQQRRDEILAKKAKLAELKEKRKARNEQGSASRPSVGVGG~E0LSIPSARNVERR
NCU09142.1	-----MQQRRDEILAKKAKLAELKRQRELRATQVNARL-SIGSAS~D0IVSPTPGRAESR
FG00428.1 	-----MQQRRDEILAKKAKLAELKRQRELRANAATA-----GRAS0-~LISPTPGRADNR
AN1454.1  	MSSAANQQRKAEILAKKAKLAELKRQRELRQKEFSQSRASTGDAS~E0VVSPVPSRSDSR
          	 :::: ***: *************.:*: *    .   .: * .. . :  * . . : *

MG04771.1 	EDLDEYINRLVGLGDRPSSTTTGGAASPGPRGSRPNSVLSAGELSNENSEYASLANGHAP
NCU09142.1	REIETLIQSLVGE-SRPVSVSTG-ANSPRLHGSRPNSVISAGETSISPSEYATPALGPIL
FG00428.1 	REIETLINSLVGD-SRPGSAVTGGPGSPAPRGSRPNSVLSAGEISNEPSEVTT--ISNIQ
AN1454.1  	AELDDLISRLVDR---PGSAAVSGADGLSRKGSRPNSVLSASQLSGDNTEALSPPTRPLS
          	 :::  *. **.    * *. .... .   :*******:**.: * . :*  : .     

MG04771.1 	AAPAAPQNLTTVPLTTIYECPPSPVK--EIFSYSKGIQTQEDWAPSSRPLELS-VSDDDE
NCU09142.1	ASSNQTTQVLSTTLTTVYECPPSPVK--EVFTYSKGVQTTDDWVPQPKPRRYS-DSEIDE
FG00428.1 	IPAQPPPSLSTAPLLTVFECPPSPVK--EVFSYSKGVQTTEEWTTPTRTRAQSLISEAED
AN1454.1  	QS-IAVQTVGTEPFVAIPELQPAPEAKPEVVTYNKGVQTDDLRQGEQD--AASVDSDAGE
          	 .      : : .: :: *  *:*  ..*:.:*.**:** :           *  *:  :

MG04771.1 	PTFTPSKR---PTRRDRDREEELRANIRREIEEELKAAQELSTDGVLKPTALTTNFPART
NCU09142.1	PPASPTKT--RVSRRLRDREEELRENIRKEIEQELKAAKELVIDGVYKP-SAAANFPTRN
FG00428.1 	VPAVTSTPGKRLSRRERDREEELRQKIREEVEEELRATKEFLSDGVVP---SAVSQPARN
AN1454.1  	TPDFKSSS-----KKQRERDEEIRKKLREEIEEELRATQESAKNESGAD--ASIRYPLRQ
          	 .   :.      :: *:*:**:* ::*.*:*:**:*::*   :        :   * * 

MG04771.1 	LTNEELKAVTASEDFVDFVDRATKVIEKALDQE-YDILTDYTLQTQENTDDREQ-GNVSG
NCU09142.1	LTDEELNAVTQSDDFMDFIDKSTKVIEKALDQE-YDILTDYTLGVNDVDEEDEQNGNTGG
FG00428.1 	WTTEELDAVAQSDDFFDFLEKSTKVIERALDQETYDILTDYALQGQDVDDDDEDSGNTGG
AN1454.1  	LDDDELKAVTSSEDFLDFVERSAKVIERALDEE-YDVLADYELGGMDAELEDDD---EHG
          	   :**.**: *:**.**:::::****:***:* **:*:** *   :   : ::     *

MG04771.1 	RGRRKIREIRQFYDERWSKKRPITSIDFSPK~FSDLVLASASKNVTAPHEPDGIVQVWNL
NCU09142.1	KGRRKVKELAQFYDERWSKKRMISSIDFSPK~FSELLLASYTKNPSAPHDPDGIVQVWNM
FG00428.1 	KGRHRIKEVAQFYDERWSKKRMISGIDFSPK~FNELLLASYTKNPTAPHEPDGLVQVWNM
AN1454.1  	KKKRGIKEVCQFWDERWSKKRMISDICFSPK0FPELVLASYTKNPSAPHEPDGLVQIWNQ
          	: :: ::*: **:******** *:.* **** * :*:*** :** :***:***:**:** 

MG04771.1 	NLHDRAEFVLHAQSDILTAKFSPFHPNLIIGGAYSGQVLLWDT---RSRSSAPVQKTPLT
NCU09142.1	HLHDRPEFVFHAQSDILTAKFSPFHPNLIIGGAYSGQVLLWDT---RAR-SAPVQKTPLT
FG00428.1 	HMQDRPEYVFTAQSDILTAKFSPYHNNLIIGGSYSGQVLLWDT---RAK-AAPVQKTPLT
AN1454.1  	HLHSRPEYVFHSTSDILTAKFSPFHPNIIVGGSYSGQVLLWDTRSSRAGGGAPVQKTPLT
          	:::.*.*:*: : **********:* *:*:**:********** ::*: ..*********

MG04771.1 	GQGHTHPVYSLDVIGTQNANNIITTSTDGVVCGWSVDMLAAPQESLMLLTPQQTK--TDD
NCU09142.1	GSGHTHPVYCIDIIGTQNANNIISCSTDGHMCGWSIDMLAQPQENLTLIAPHPSR--TED
FG00428.1 	GYGHAHPIYSVDIVGTQNANNIISCSTDGVVCGWTMDVFAQPQELLELKNPSQAKVAVED
AN1454.1  	GAGHTHPVYSISIVGTQNAHNIITASTDGVVCGWTVDMLSQPQEYLELSTPPPSK--TED
          	* **:**:*.:.::*****:***: **** :***::*::: *** * *  *  ::  .:*

MG04771.1 	LSPTCMAFPQADPTYFLVGSEEGVIYPCHRYDRAGAKAGVDKNVSYRGHSAPVRSVDFHS
NCU09142.1	MSPTCMAFPQADPTFFLVGTEEGSIYPCHRYDRAGAKAGVDARVSYRGHAAPVMAVDFHP
FG00428.1 	VSPTCLSFPDTDPTFFLVGSEEGTIFPCHRYDRAGAKAGVDKKISYKGHTAPVMSVDFHP
AN1454.1  	LAPTTISFPQSDPTFFIVGTEEGGIYPCHRYDRAGAKAGTDHRLAYRGHAAPIMSTAFHP
          	::** ::**::***:*:**:*** *:*************.* .::*:**:**: :. **.

MG04771.1 	NLGPVDFGDLVLSSSLDWSAKLWKVRPPGGIGTAGLLSSN------AEVAVSPLMEFARE
NCU09142.1	ARGVVDLGDLVLSASLDWSVKLWKARAPAATSTVVTTGGGINVPGQEHNGVQPLIDFVRE
FG00428.1 	ARGPVDLGDLVISSSLDWSVKLWKVRAPAATSTIGSGDG----------TIAPLIDFVRE
AN1454.1  	ARGPVDLGDLMLSSSLDWSVKLWRIRPPAATAPAATSGIT------DTQVVTPILDINRE
          	  * **:***::*:*****.***: *.*.. ..    .            : *:::: **

MG04771.1 	DIVYDAAWSPLRPGVFSLVDGAGYLEIWDLTVETEEPVARIQPNARPGAR---VASLNKV
NCU09142.1	DVVYDVAWSPVKPGVFSLVDGAGWLELWDITVETEEPVARISPSPRKDGRPMLSKSLNKV
FG00428.1 	DVVYDAKWSPVKPNIFALVDGAGWLELWDIAVETEEPVSKISPSSRKDGRTMLSKSLNKV
AN1454.1  	DVVYDARWSPHRPGVFSLVDGAGNLEVWDLYTDTEVPAVRTTPS--KGRAGVLSRSLNKV
          	*:***. *** :*.:*:****** **:**: .:** *. :  *.   .       *****

MG04771.1 	AWERSEGKRLATGGIAGALTVFEVGPDLGGK-EGIKSDEWAAVKKLISKLELDAKGPVNG
NCU09142.1	KWERTEGKRLATGGIDGTVTMFEVGPDLGGK-EDLKNEEWQSVKKLVQKAEHMISLKEAQ
FG00428.1 	AWEPNDGKRLATGGIDGSLTVFEVASGLGGK-ESLKNEDWANVKKLVNRLEAVGSNGALI
AN1454.1  	AWEEREGRRLATGGLDGVVTVFEVGRGLSGTPDDVPSEEWTGMKRLVGKLEQKDKDRMS-
          	 **  :*:******: * :*:***. .*.*..:.: .::*  :*:*: : *   .     

MG04771.1 	VSGLRN
NCU09142.1	EK----
FG00428.1 	V-----
AN1454.1  	------
          	
```
